# MDL-28170 Has No Analgesic Effect on CCI Induced Neuropathic Pain in Mice 

**DOI:** 10.3390/molecules15053038

**Published:** 2010-04-27

**Authors:** Nurcan Üçeyler, Lydia Biko, Claudia Sommer

**Affiliations:** Department of Neurology, University of Würzburg, Josef-Schneider-Str. 11, 97080 Würzburg, Germany

**Keywords:** calpain, neuropathic pain, MDL-28170, chronic constriction nerve injury (CCI)

## Abstract

The calpain inhibitor MDL-28710 blocks the early local pro-inflammatory cytokine gene expression in mice after chronic constriction nerve injury (CCI). One-hundred-thirteen wild type mice of C57Bl/6J background received CCI of the right sciatic nerve. Mechanical paw withdrawal thresholds and thermal withdrawal latencies were investigated at baseline and at 1, 3, and 7 days after CCI. Three application regimens were used for MDL-28170: a) single injection 40 min before CCI; b) serial injections of MDL-28170 40 min before and up to day three after CCI; c) sustained application via intraperitoneal osmotic pumps. The control animals received the vehicle DMSO/PEG 400. The tolerable dose of MDL-28170 for mice was 30 mg/kg body weight, higher doses were lethal within the first hours after application. Mechanical withdrawal thresholds and thermal withdrawal latencies were reduced after CCI and did not normalize after single or serial injections, nor with application of MDL-28170 via osmotic pumps. Although the calpain inhibitor MDL-28170 inhibits the early local cytokine upregulation in the sciatic nerve after CCI, pain behavior is not altered. This finding implies that local cytokine upregulation after nerve injury alone is only one factor in the induction and maintenance of neuropathic pain.

## Introduction

Nerve injury in mice is associated with pain behavior and is paralleled by an increase in local cytokine gene expression. Chronic constriction nerve injury (CCI) is a frequently used neuropathic pain model, which leads to an early increase in local gene expression of the pro-inflammatory cytokines tumor necrosis factor-alpha (TNF) and interleukin (IL-)1β in the lesioned sciatic nerve already within the first hour [1]. Nerve injury results in cellular calcium influx, which in turn triggers downstream mediators and enzyme systems like the caspases and calpains. Calpains are a family of calcium dependent cysteine proteases and are involved in cellular processes like cytoskeletal protein cleavage, apoptosis, cell proliferation and differentiation, and synaptic plasticity [2,3,4,5]. Calpains are ubiquitously expressed and act within the very first hours after nerve injury; physiologic levels are reached within few hours [6]. Furthermore, calpains trigger the expression of mediators of Wallerian degeneration like IL-1β. In turn, the inhibition of calpains protects axons from trauma induced damage and reduces pain [7,8]. Thus, calpains may be among the early triggers of neuropathic pain. 

**Figure 1:**
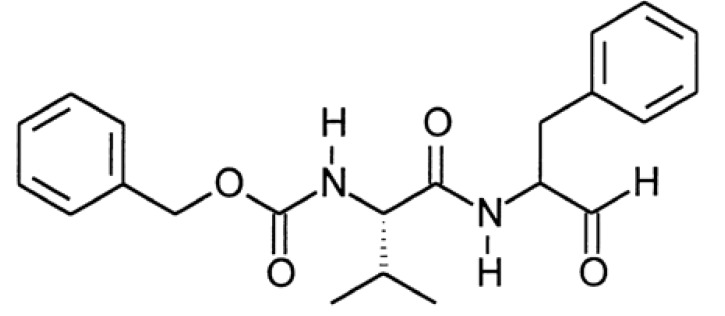
Chemical structure of MDL 28,170 (*N*-benzyloxycarbonylvalylphenylalaninal).

MDL-28170 (carbobenzylzoxy-Val-Phe-H) is a central nervous system penetrating calpain inhibitor (Figure 1) that passes the blood–brain barrier and inhibits brain cysteine protease activity after systemic application. Kunz et al. described anti-inflammatory and analgesic effects of MDL-28170 in the zymosan-induced paw inflammation model in rats [7]. We previously showed that the systemic pre-lesional application of the calpain inhibitor MDL-28170 attenuates the early local pro-inflammatory cytokine response after CCI [1].

## Results and Discussion

### MDL-28170 dose in mice

Following the manufacturer`s (Calbiochem, Schwalbach, Germany) instructions we first used dimethylsulfoxide (DMSO) to dissolve MDL-28170 and in control mice, however, this substance in combination with MDL-28170 was not well tolerated and the majority of the experimental animals died within few hours after injection. We then used DMSO/PEG 400 (1:1) instead of DMSO alone (personal communication with E. Niederberger, Pharmazentrum Frankfurt, Institut für Klinische Pharmakologie, Klinikum der Johann Wolfgang Goethe-Universität Frankfurt, Germany), which was better tolerated by the mice. We found that a dose of 30 mg/kg body weight per intraperitoneal (i.p.) injection was well tolerated by the mice.

### MDL-28170 does not normalize paw withdrawal latencies and thresholds after CCI

**Figure 2:**
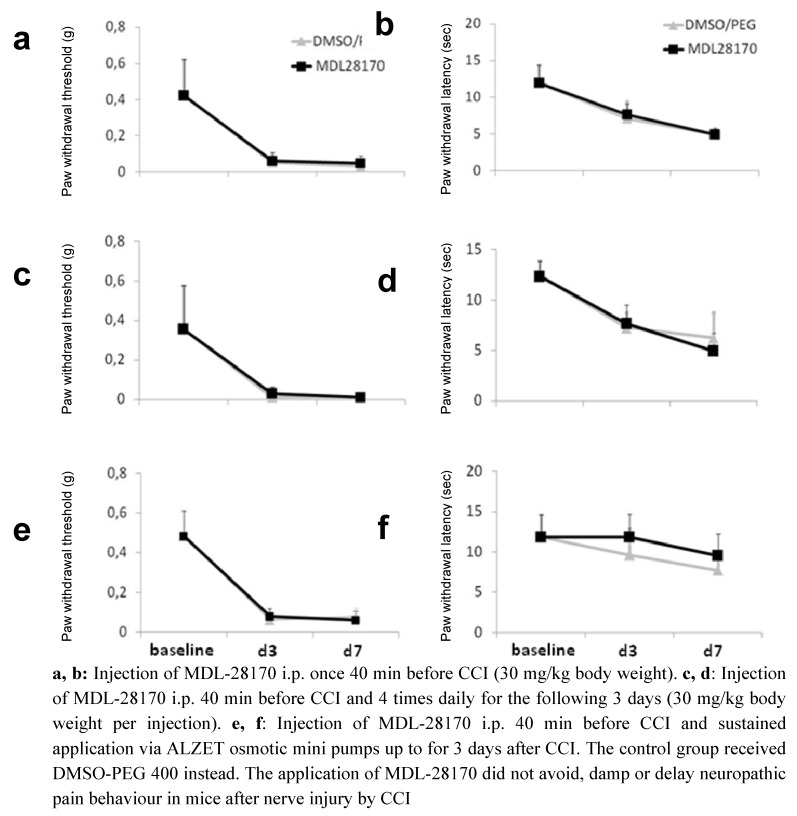
Paw withdrawal thresholds to mechanical stimuli (a, c, e) and paw withdrawal latencies to thermal stimuli (b, d, f) in mice after chronic constriction injury (CCI) and different application regimes of MDL-28170.

After CCI mice developed reduced paw withdrawal latencies and thresholds (Figure 2). The application of MDL-28170 40 min before and immediately after surgery did not prevent the development of pain behavior in mice at day 3 and day 7 (Figure 2a, b). The injection of vehicle (DMSO/PEG 400) had no effect on pain behavior. 

To achieve more consistent levels of MDL-28170 in the organism we injected the animals four times a day for 3 days, starting with an injection 40 min before CCI. Each injection consisted of 30 mg/kg body weight. Again, the induction of pain behavior was not delayed or reduced (Figure 2c, d). 

We then implanted ALZET mini pumps filled with MDL-28170 immediately after CCI for sustained MDL-28170 application and tested the mechanical and thermal withdrawal thresholds and latencies. In this experiment no normalization was found for mechanical withdrawal thresholds after CCI and under MDL-28170 tretment (Figure 2e); however, a trend was found for a delay in reduction of paw withdrawal latencies in the MDL-28170 treated mice (Figure 2f; n.s.). At day 7 the MDL-28170 and the DMSO/PEG 400 group showed reduced withdrawal latencies.

### MDL-28170 does not changeTNF gene expression in the central nervous tissue of mice after CCI

**Figure 3:**
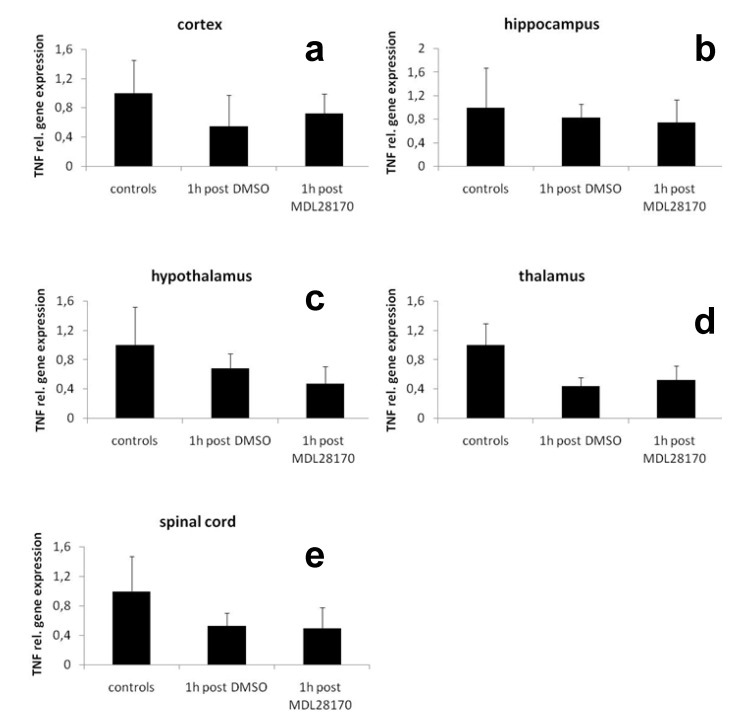
Relative gene expression of TNF in mouse a) cortex, b) hippocampus, c) hypothalamus, d) thalamus, e) spinal cord 1 hour after CCI and after application of MDL-28170 or DMSO.

To investigate if the application of MDL-28170 changes pro-inflammatory cytokine expression in the central nervous system of mice after CCI we analyzed the mRNA expression of TNF in cortex, hippocampus, hypothalamus, thalamus, and spinal cord of mice 1h after CCI. No difference was found for the expression of TNF in these tissues compared to naïve wildtype mice (Figure 3a-e). 

In summary, our results show: 1) that the calpain inhibitor MDL-28170 in the doses and types of application used here is not capable of preventing, reducing or delaying neuropathic pain behaviour in mice after nerve injury by CCI; 2) that MDL-28170 is poorly tolerated by mice; 3) that although MDL-28170 attenuates the local pro-inflammatory response [1,7] this alone seems not sufficient to avoid pain behavior upon mechanical and thermal stimuli. 

MDL-28170 is mainly used in *in vitro* experiments (for example [9,10,11]). *In vivo* experiments were mostly performed in rats [7,12,13,14,15,16]. A literature search (PubMed, through December 2009) revealed that only in few studies MDL-28170 was applied in mice [1,17,18,19,20]. In our own previous studies we injected MDL-28170 as a single application i.p. 40 min before CCI at a dose of 30 mg/kg body weight and showed that the early upregulation of local TNF and IL-1β gene expression in injured sciatic nerve one hour after CCI was attenuated. Mice were sacrificed one hour after CCI [1,17]. In the study by Crocker *et al*. the local and sustained application of MDL-28170 *via* Alzet osmotic mini pumps in a mouse model of Parkinson`s disease abolished locomotor deficits and markers of striatal postsynaptic activity were normalized. The osmotic pumps were filled with 160 µM MDL-28170 and were implanted into the right lateral ventricle of mice; the animals were sacrificed after three weeks [18]. In the study of Wang and collegues MDL-28170 was injected i.p. once in a dosage of 30 mg/kg body weight and the protective effect against thioacetamide-induced acute liver failure was investigated in the “surviving mice” after three days [20]. In mice deficient of cardiotrophin-1 (a member of the IL-6 family with hepatoprotective effects) MDL-28170 injection protected against Fas-induced liver injury [19]. The treatment regime was MDL-28170 60 µg/g body weight 1 hour after the injection of the agonistic anti-Fas monoclonal antibody Jo-2; the animals were monitored up to 30 hours. 

The reported short survival times in these studies may indicate that MDL-28170 is not very well tolerated by mice when administered systemically. The effects of MDL-28170 have been examined in several animal studies also in rats or gerbils. On the field of ischemic stroke MDL-28170 was applied immediately after hypoxic exposure in neonatal rats (24 mg/kg; i.p. as an initial dose, followed by 12 mg/kg every 4 hours for a total dose of 60 mg/kg over 12 hours after ischemic brain lesion). The animals were sacrificed 24 hours after injury. In this study MDL 28170 decreased the number of necrotic and apoptotic cells in distinct brain areas [15]. In one study using a gerbil model of global cerebral ischemia MDL-28170 was injected i.p. at a dose of 50 mg/kg body weight at four time points after ischemia starting 0.5 and 3 h after reperfusion. MDL-28170 protected cortical neurons from ischemic damage even if the treatment was delayed until 3 hours after reperfusion [21]. 

MDL-28170 had neuroprotective effects in a rat model of fluid percussion injury of the brain with a therapeutic time window for up to 4 hours after a single administration of MDL-28170 post injury [12]. In a rat model of contusive spinal cord injury the combined application of MDL-28170 intravenously and daily i.p. resulted in improvement of functional and pathological outcome measures; rats were investigated up to day 42 after injury [16]. Similarly, intravenous injection of 10 mg/kg body weight MDL-28170 after injury for 1 week every 24 hours prevented neuronal cell death and improved motor disturbance after compression-induced spinal cord injury in rats [13]. Buki *et al*. showed that the single-dose pre-injury administration of MDL-28170 into the tail vein of rats 30 min before injury (30 mg/kg body weight) attenuates traumatically induced axonal injury [14]. Rats were sacrificed 120 min after injury. In another study the neuroprotective effect of MDL-28170 was investigated in a rat model of status epilepticus [22]. MDL-28170 was applied i.p. in a dose of 50 mg/kg body weight 30 min before and 1 hour after pilocarpine injection (*i.e.,* induction of seizure). Rats were sacrificed 3 days after seizures and was shown, that MDL-28170 partially attenuated neuronal death in the hippocampus [22]. 

The only study investigating the effect of MDL-28170 on pain behavior was performed by Kunz and collegues [7]. In the zymosan-induced paw inflammation model of rats MDL-28170 was injected i.p. 20 min before zymosan treatment (12.5 mg/kg or 25 mg/kg) or was given peridurally at a dose of 1 mg/kg body weight [7]. Drug treatment in any regime normalized paw withdrawal latencies. Animals were examined up to 96 hours after MDL-28170 injection. 

In our study we did not achieve normalization of paw withdrawal latencies and thresholds after CCI in mice using three different treatment regimes with MDL-28170. Although MDL-28170 attenuates the local increase in TNF and IL-1β gene expression in the sciatic nerve [1], this alone seems not sufficient to attenuate pain behavior in a neuropathic model. This finding may be due to the fact that nerve lesion leads to a wide range of changes in the gene expression of different cytokines and their interactions. There are some limitations to our study. We have not investigated the gene expression of TNF and IL-1β at later time points and under the different treatment regimens. Thus, it is possible, that MDL-28170 only has a very early effect on TNF and IL-1β gene expression (*i.e., *within the first hour after nerve lesion), but does not hinder cytokine gene expression at later time points. Since the earliest time point for reproducible behavioral testing in mice after CCI surgery at day 3, we cannot exclude that the local cytokine response might have increased by then. Furthermore, the dose applied may not have been sufficient. However, higher doses were not tolerable. Other substances might be used as vehicles instead of DMSO alone or DMSO/PEG 400 to dissolve MDL-28170, possibly with better tolerability (e.g. Krebs` Ringer solution [18], 0.6% carboxymethyl cellulose [21], peanut oil [15]). The use of normal saline is inadequate [22], since MDL-28170 does not dissolve in solvents on water basis. One has to keep in mind that we investigated the effect of MDL-28170 only after CCI - a neuropathic pain model. Given the results by Kunz *et al*. [7] the substance may be more effective in inflammatory pain models.

## Experimental

### Animals

Eight to ten weeks old female wild type mice (n = 113; 18-23 g body weight) of C57Bl/6J background were used. Mice were purchased from Charles River Laboratories (Sulzfeld, Germany) and were held at the animal facilities of the Department of Neurology of the University of Würzburg under standard conditions with food and water access *ad libitum*. All experiments were approved by the Bavarian State authorities.

### Surgery

Animals received a CCI at the right sciatic nerve followed the original description [23] with slight modifications [24]. Briefly, the sciatic nerve was exposed under intraperitoneal (i.p.) barbiturate narcosis (Narcoren®, 50 mg/kg body weight). Three ligatures (7-0 prolene) were placed around the nerve proximal to the trifurcation with a distance of one mm between each ligature. The ligatures were loosely tied until a short flick of the ipsilateral hind limb occurred. 

### Behavioral testing

Mechanical paw withdrawal thresholds (von Frey test) and thermal paw withdrawal latencies were investigated at baseline (*i.e.,* once a day on two days before CCI) and at days 1, 3, and 7 after CCI. The investigator was not aware of the pharmacological treatment the animals had received. The *von Frey test* based on the up-and-down-method as previously described [25]. Animals were placed in Plexiglass cages on a wire mesh and the plantar surface of the hind paws was touched with a von Frey filament. The measurements started at a hair value of 0.69 g. When the animal withdrew its hindpaw upon application of mild pressure the next finer von Frey filament was used. If the animal did not react to the stimulus, the next stronger von Frey filament was used. Each hind paw was tested three times. The 50% withdrawal threshold (*i.e.,* force of the von-Frey hair to which an animal reacts in 50% of the administrations) was recorded. The *thermal paw withdrawal latencies *were investigated applying the method of Hargreaves [26] and using a standard Ugo Basile Algesiometer (Comerio, Italy). Mice were placed on a glass surface surrounded by Plexiglass cages. A radiant heat source was positioned under one hind paw and the time until a withdrawal reaction occurred was measured. The time limit for heat application was 15 sec at maximum to avoid burning damage. Each hind paw was tested three times with a minimum interval of 2 min between the measurements to avoid habituation. The investigator was blinded as to the treatment of the mice. 

### Pharmacological treatment

We used the calpain inhibitor III MDL-28170 (Calbiochem, Schwalbach, Germany) for our experiments. MDL-28170 was dissolved in DMSO/PEG 400 (1:1). DMSO/PEG 400 was used instead of DMSO alone to reduce mouse lethalty after injection. Treatment was applied by an investigator blinded to the substance. 

### Treatment regimes and dosages

Single injection i.p.: We previously showed that the single shot i.p. treatment of mice 40 min before CCI using MDL-28170 inhibits early upregulation of pro-inflammatory cytokine gene expression in the sciatic nerve [1]. To investigate if this single shot injection also reduces pain behavior after CCI we treated mice with 30 mg/kg body weight i.p. 40 min. before CCI. Control animals received equivalent volumes of DMSO/PEG 400 (1:1). 

Serial injections i.p.: To increase the amount of MDL-28170 and prolong its possible analgesic effects we treated mice 40 min. before CCI and four times per day for three days after CCI with 30 mg/kg body weight each. The cumulative dose per kg body weight of MDL-28170 applied per mouse was 390 mg. 

Osmotic pump: To achieve sustained drug release we injected 30 mg/kg body weight per mice i.p. 40 min before CCI and implanted Alzet osmotic pumps (model 1003D, Alzet, USA) i.p. immediately after CCI. Pumps for 3 days of drug release were used and the cumulative dose of MDL-28170 applied per kg body weight per mouse was 390 mg. 

### Relative gene expressin of TNF in the central nervous system under MDL-28170 treatment

In previous pilot experiments we investigated the relative gene expressin of TNF in the central nervous system of mice after CCI. Ten wildtype mice of C57Bl/6J background were treated with MDL-28170 or DMSO 40 min before CCI at a dosage of 30 mg/kg body weight. One hour after CCI cortex, hippocampus, hypothalamus, thalamus, and the spinal cord were dissected and immediately shock-frozen in liquid nitrogen before further processing. RNA extraction, reverse transcription and quantitative real-time PCR (qRT-PCR) were performed as previously described [1]. 

## Conclusions

CCI leads to an early increase in local pro-inflammatory cytokine expression associated with pain behavior in mice. The calpain system is involved in triggering cytokine production in the very early few hours after nerve injury and may be of key importance also in the induction of pain behavior. The application of the calpain inhibitor MDL-28170 has proven to attenuate the early pro-inflammatory response and to improve thermal latencies in rats, however, the i.p. application of MDL-28170 (single injection, multiple injections, osmotic pump) before and after CCI does not normalize withdrawal latencies and thresholds in mice after CCI. MDL-28170 is of limited importance in the investigation of neuropathic pain in mice after CCI. 
